# Wrist Circumference and Frame Size Percentiles in 6-17-Year-Old Turkish Children and Adolescents in Kayseri

**DOI:** 10.4274/jcrpe.4265

**Published:** 2017-12-15

**Authors:** Ahmet Öztürk, Betül Çiçek, M. Mümtaz Mazıcıoğlu, Gökmen Zararsız, Selim Kurtoğlu

**Affiliations:** 1 Erciyes University Faculty of Medicine, Department of Biostatistics and Medical Informatics, Kayseri, Turkey; 2 Erciyes University Faculty of Health Sciences, Department of Nutrition and Dietetics, Kayseri, Turkey; 3 Erciyes University Faculty of Medicine, Department of Family Medicine, Kayseri, Turkey; 4 Erciyes University Faculty of Medicine, Department of Pediatric Endocrinology, Kayseri, Turkey; 5 Turcosa Analytics Solutions Ltd. Co, Erciyes Teknopark 5, Kayseri, Turkey

**Keywords:** Adolescents, anthropometry, children, frame size, growth percentiles, wrist circumferences

## Abstract

**Objective:**

The aim of the current study was to provide wrist circumference (WrC) and body frame size (height/WrC) percentile values in Turkish children and adolescents aged 6-17 years.

**Methods:**

In this cross-sectional study, the data of “Determination of Anthropometric Measures of Turkish Children and Adolescents” (DAMTCA II) study in Kayseri/Turkey were used. A total of 4330 observations were recorded (1931 boys, 2399 girls). The WrC and frame size reference values were produced with generalized additive models for location, scale and shape.

**Results:**

The WrC percentiles (3rd-97th) were calculated. The frame size (height/WrC) was estimated as small, medium, and large (<15^th^, 15-85^th^, and ≥85^th^ percentiles, respectively). For both genders, WrC linearly increased with age (13.0-16.8 cm for boys and 12.5-15.5 cm for girls). In boys and girls, the mean ± standard deviation of WrC is 13.00±0.89 cm and 12.48±0.93 cm (6 years) and increases to 16.83±1.16 and 15.58±0.86 cm (17 years), respectively. The WrC values in all age groups were higher in boys compared with girls. The increment in frame size from 6 to 17 years were 1.25 cm in boys and 0.85 cm in girls.

**Conclusion:**

WrC is a simple, easy-to-detect anthropometric index which is not subject to measurement errors. Additionally, WrC can be used both to decide about frame size and to determine metabolic risks related to obesity. We consider that this easy-to-get anthropometric index can be used both in screening procedures and clinical assessment procedure for obesity-related metabolic consequences.

**What is already known on this topic?**

Studies on reference values for wrist circumference and their use in assessing cardio-metabolic risks are limited.

**What this study adds?**

This is the first study on wrist circumference in Turkish children and adolescents and was conducted on a quite large sample. The study also included estimation of frame size based on wrist circumference.

## INTRODUCTION

Health professionals frequently use direct anthropometric measurements and indices derived from these measurements as tools to determine cardiometabolic risks and obesity. Definition of nutritional status, assessment of growth and development status, evaluation of differences in body proportions between populations, as well as contribution to diagnosis and treatment are some of these uses (1,2). Although waist circumference (WC) is the most frequently used index to assess nutritional, metabolic, and cardiovascular disorders, mid-upper arm, wrist, and neck circumferences may also be used (3,4).

Wrist circumference (WrC) is a new and possibly promising measurement to assess body frame size. A recent cross-sectional study suggests that WrC may also be associated with insulin resistance in obese children and adolescents (5). Most recently, in an 8.8-year follow-up study in a sample of 9330 adult Iranians, WrC was reported to be a significant predictor of diabetes in both genders (6). Although there is no established consensus on the routine use of WrC for clinical purposes as yet, there are studies suggesting that WrC may be a promising measurement.

The aim of this current study was to provide reference values for WrC and height/wrist ratio to assess body frame size in Turkish children and adolescents. To our knowledge, this will be the first study in the Turkish population on this issue. Additionally, producing frame size percentiles in this study would provide the assessment of WrC adjusted for height.

## METHODS

### Study Design and Sampling

We used the data of the “Determination of Anthropometric Measures of Turkish Children and Adolescents (DAMTCA II)” study, which was conducted during October 2007 and April 2008. The DAMTCA II study was performed in Kayseri province which has more than 1,200,000 inhabitants and which is a leading industrial and trade center in Turkey. A total of 4330 observations were recorded (1931 boys, 2399 girls). The sampling design of the study was cross-sectional in which multi-stage probability sampling was performed (4).

Of the 708 schools in Kayseri, 17 primary, secondary, and high schools were randomly selected to recruit children and adolescents aged 6-17 years. Chronological age was calculated as the decimal age by subtracting the observation date from the birth date. Each year elapsed from their birthdates was noted as one age (e.g. 7.00-7.99 is accepted as 7 years old). The study protocol was approved by the Ethics Committee of Erciyes University and by the administration of the local educational authority (approval number: 2008/28, date: 08.01.2008). Parents’ written consent was obtained prior to the study, and the procedures were in accordance with those outlined by the Declaration of Helsinki.

### Data Collection and Questionnaire

**Anthropometric indices:** All measurements were performed twice by well-trained health professionals and the arithmetic mean was recorded for evaluation.

**Wrist circumference:** WrC was measured with children/adolescents in a seated position using a tension-gated tape measure positioned over the Lister tubercle of the distal radius and over the distal ulna. The Lister tubercle, a dorsal tubercle of the radius, can be easily palpated at the dorsal aspect of the radius around the level of the ulna head, about 1 cm proximal to the radiocarpal joint space. A tension-gated tape measure was used to ensure equivalent tape pressure between subjects (5).

**Height:** Height was measured with a portable Seca stadiometer sensitive to changes up to 1 cm. Daily calibration was performed to the portable devices. Measurements were done with subjects barefoot, the heels, hips, and shoulders touching the stadiometer, and the head in neutral position with eyes gazing forward.

**Grant index:** Height (cm)/WrC (cm) (skeletal value) 1,7).

**Questionnaire:** The survey was based on a questionnaire sent home prior to evaluation and collection of anthropometric data from participating children/parents and adolescents.

### Statistical Analysis

Outliers were examined first by checking the discontinuities in age-related WrC z scores plot, also z<-10 standard deviation scores (SDS), and z>6 SDS liberal cut-off values were applied for automatic outlier detection (8). The remaining 4330 observations (1931 boys, 2399 girls) were randomly split into training and test sets (70%, 30%). The training set was used to fit the models (for each distribution and each gender), and the test set was applied to avoid overfitting, validate models, and to choose the convenient distribution.

Generalized additive models for location scale and shape (GAMLSS) were used to fit the age-related WrC model (9). Maximum penalized likelihood estimation was used to fit the model using Rigby and Stasinopoulos algorithm and Fisher scoring procedure. Box-Cox power exponential (BCPE), Box-Cox t (BCT), and Box-Cox Cole and Green (BCCG) distributions (distributions of LMS, LMST, and LMSP methods, respectively) were used to fit models, and cubic splines were used as smoothing functions. Each gender was modelled separately. The GAMLSS package (version 4.1-1) of R 2.14.0 program (www.r-project.org) was employed to fit our data.

For BCPE distribution modeling of boys, we followed the three-step optimization procedure of Rigby and Stasinopoulos (10) and the Generalized Akaike Information Criterion (GAIC#3) for model selection. In the first step, identity link functions for μ and ν, log link functions for σ and τ were chosen. An initial age transformation was chosen as x=ageλ=2.5 after a grid search of λ between -3 to 3 in steps of 0.5. In the second step, initial degrees of freedom of all four parameters were set to 1, and values of df (μ), λ, and df (σ) were optimized, respectively. For df (μ) and df (σ), we made a search between 1 to 20 in steps of 1 and for λ between 2 to 3 in steps of 0.01. Starting model led to BCPE [3,1, df (ν), df (τ), 2.53] and fitted to all combinations of df (ν) and df (τ) ranging between 0 to 9 and 0 to 4 in steps of 1, respectively. In the last step of the procedure, fine tuning was used for the model BCPE (3, 1, 1, 0, 2.53) with changing values of df (σ), df (μ), df (ν), df (τ), and λ. We obtained BCPE (2.9, 1.0, 0.9, 0.2, 2.51) as the final model.

The same procedure was performed for girls and for BCT distribution models. For BCCG distribution, similar but a little different procedure was performed because of the absence of τ parameter. The difference is that values of df (μ) and λ were optimized first and combinations of df (σ) and df (ν) were searched next in a second step for same link functions (for μ, σ and ν). Finally, BCPE (2.9, 1.0, 0.9, 0.2, 2.51), BCT (2.8, 1.0, 0.9, 0.0, 2.20), and BCCG (2.9, 1.0, 1.0, 2.30) models for boys and BCPE (3.6, 1.1, 1.0, 0.6, 1.55), BCT (8.4, 1.0, 1.0, 0.1, 0.80), and BCCG (9.1, 0.8, 1.8, 1.20) for girls were obtained as optimum models for LMSP, LMST, and LMS methods, respectively. Based on the minimum GAIC#3 values, we decided to fit the LMSP method to construct percentile curves for both boys and girls.

A similar procedure was applied to model age-related frame size percentiles. Optimum models were obtained as BCPE (1.40, 4, 1, 1) for boys and BCCG (-0.75, 7, 1, 1) for girls. Differences between both genders in each age were assessed by independent samples t-test. Two-tailed p-values of <0.05 were considered statistically significant.

## RESULTS

Table 1 shows the mean and percentile values for WrC in 6-17-year-old Turkish boys and girls. For both genders, WrC linearly increases with age (13.0 to 16.8 cm for boys and 12.5 to15.5 cm for girls, respectively for 6 and 17 years). In boys and girls, the mean ± standard deviation of WrC is 13.00±0.89 cm and 12.48±0.93 cm (6 years) and increases to 16.83±1.16 and 15.58± 0.86 cm (17 years), respectively. The WrC values in all age groups were higher in boys when compared with girls. In boys, the 50^th^ percentile ranged from 12.92 cm (at age 6) to 16.85 cm (at age 17) and in girls, the 50^th^ percentile ranged from 12.41 cm (at age 6) to 15.54 cm at age 17.

**Figure 1:**
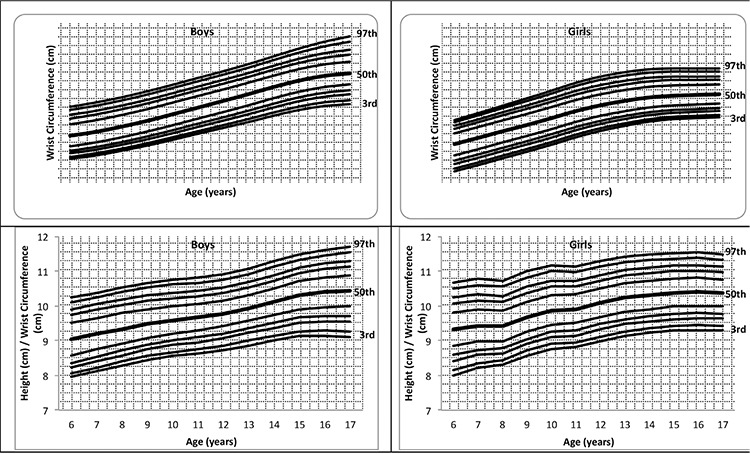
The distribution of wrist circumference and frame size in boys and girls aged 6-17 years with fitted percentile curves

Figure 1 shows the fitted percentile curves of frame size (height/WrC) and WrC (3^rd^, 5^th^, 10^th^, 15^th^, 25^th^, 50^th^, 75^th^, 85^th^, 90^th^, 95^th^, 97^th^) of related models for 6-17-year-old boys and girls. WrC steadily increases until 17 years in boys, while it makes plateau after age 14 years in girls.

**Figure 2:**
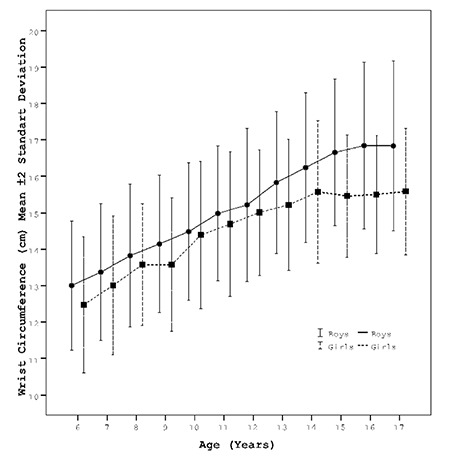
The change in wrist circumference between each gender through 6-17 years (mean ± 2 standard deviation)

Table 2 shows the percentile values for frame size (height/WrC) of 6-17-year-old Turkish boys and girls. In Table 3, the age- and gender-related frame size is shown according to its small, medium, and large percentiles. The three-frame size categories are determined according to certain percentiles: small ≤15th percentile, medium >15^th^ percentile and <85^th^ percentile, and large ≥85th percentile) (1). The increment in mean frame size from 6 years to 17 years was 1.26 cm in boys and 0.85 cm in girls. The change in WrC distribution through 6 to 17 years for each gender is shown in Figure 2 to demonstrate the difference between each gender and age-related increment. In Figures 3A and 3B, we compared the 50th WrC percentiles in the available three different studies to reveal geographic differences (2,11).

## DISCUSSION

Body circumferences are recommended for use in the clinical evaluation of nutritional and cardiometabolic disorders as a measure of compartmental body fat content or as an index of body fat distribution. As a novel clinical measure, we aimed to design a frame to calculate percentiles of WrC and also indices in which WrC is an important determinant to assess body frame. We found that WrC was higher in boys than in girls aged 6-17 years. The gender difference is observed to be about 0.5-1.0 cm from 6 to 12 years. Later, through 12 to 18 years, the gender difference gradually increases to 1.0-2.0 cm in favor of boys. (Table 1, Figure 2). Collinearity of WrC and WC is the most promising character of WrC, since it can be measured very easily. Additionally, WrC measurement is free from distracting factors, such as respiration and abdominal distension that can alter the reproducibility of WC.

In obese Italian children, hyperinsulinemia-related increase in free insulin-like growth factor-1 (IGF-1) levels was considered to enhance bone development and consequently lead to increase in WrC (5). Thus, WrC measurements were suggested to contribute to the assessment of hyperinsulinemic obesity, in addition to calculating frame size (frame size=height/WrC). The initial recommendation for WrC as a risk factor for metabolic disorders in adult population depends on a recent prospective study (6), although the first study in adolescents for WrC references was conducted in Bolivian 12-18-year-old adolescents (2). This present study is the third study on WrC and has been conducted on a large sample size with a broad age spectrum.

According to an adult study in the USA and Canada, there is a statistically significant positive association between WrC and diabetes (odds ratio=1.3, p=0.006). In this study, diabetic women also had larger neck, bust, and wrist circumferences, when adjusted for age and body mass index (2). In another pilot study, non-diabetic Italian adolescent athletes with a positive family history demonstrated statistically significant higher WrC values when compared to athletes with a negative family history and the difference was more significant in males (13). We consider that our WrC data can be used to investigate the possibility of a similar association of cardiometabolic risk in our children and adolescents.

As mentioned above, WrC measurement can be applied easily and has relatively low measurement errors. It is a simple, easy-to-detect anthropometric index, and it is not subject to measurement problems due to estimation of precise anatomic definition or to effects such as respiratory movements in WC measurements (14).

Another contribution of this present study is producing frame size as well as WrC references. Frame size classifies the skeletal structure as small, medium, and large, a characteristic that provides information in assessment of body composition. Fat mass, fat-free mass, and bone mass compositions are used in assessment of cardiometabolic risk. Frame size may contribute significantly to assess the level cardiometabolic risk since individuals with similar body fat content or body fat distribution may have different risk levels according to their frame size (15). The calculated cut-offs for frame size in the current study may then be used in assessment of cardiometabolic risk or obesity for both genders in children and adolescents. WrC is defined as an inexpensive, non-invasive, safe, inner-, inter-observable valid measure (16). Additionally, WrC is a measurement to predict insulin resistance (16). Finally, to the best of our knowledge, our study has been conducted on the largest sample size to date.

### Study Limitations

On the other hand, the cross sectional study design and relatively underrepresenting total country population may be the limitations of this present study.

## CONCLUSION

In conclusion, we may consider that WrC is a well-known anthropometric, however a novel clinical measure which can also be used to assess body frame size that may reflect cardiometabolic risk factors. This is the first comprehensive study, providing both WrC and frame size percentiles in Turkish children and adolescents. In short, WrC measurement is an easy to apply method with a relatively low risk of faulty measurement. However, studies on its use and applicability as a reference or risk factor are limited. Finally, WrC measurements and estimation of frame size have also the potential to be used in ergonomics for product design, human-machine harmony, and for analysis of physical environment for health.

## Figures and Tables

**Table 1:**
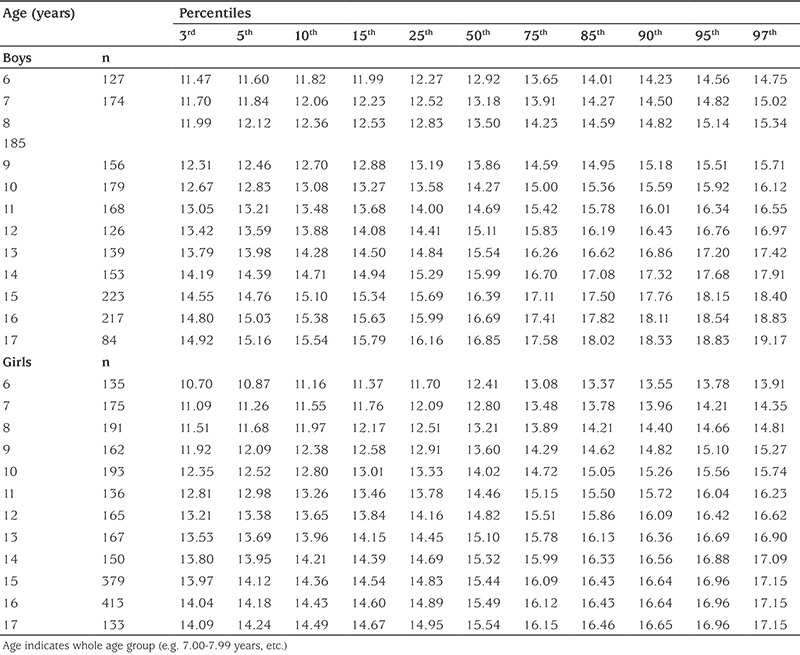
Age-related wrist circumference (cm) percentiles of 6-17-year-old Turkish boys and girls

**Table 2:**
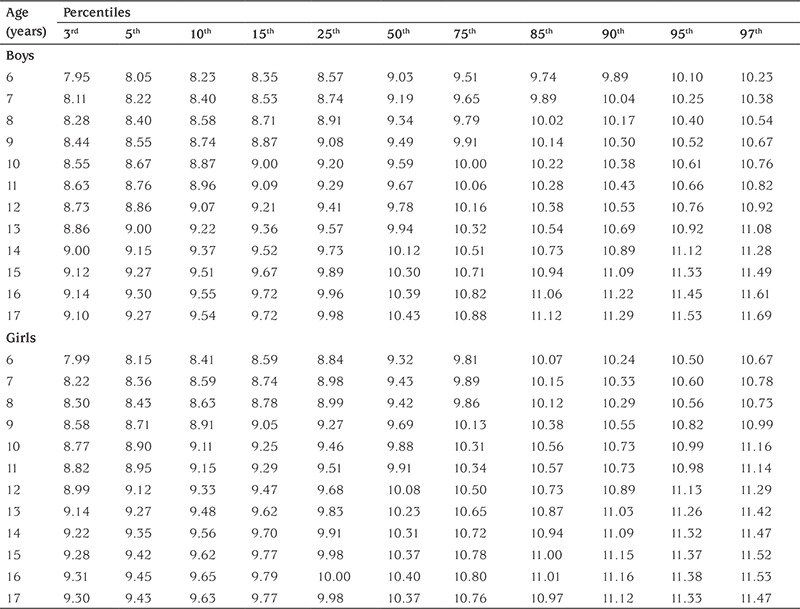
Age-related frame size [height (cm)/wrist circumference (cm)] percentiles of 6-17-year-old Turkish boys and girls

**Table 3:**
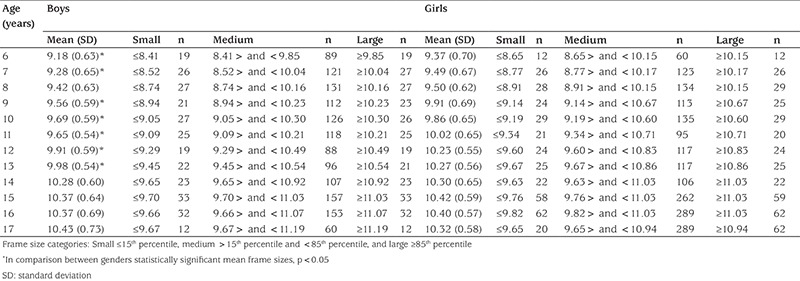
The frame size categories [height (cm)/wrist circumference (cm)] for each gender

**Figure 3A:**
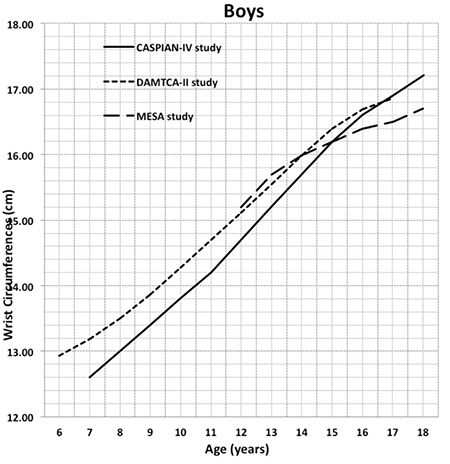
Comparison of 50^th^ percentiles of boys Wrist circumferences in Determination of Anthropometric Measures of Turkish Children and Adolescents-II, childhood and adolescence surveillance and prevention of adult non-communicable disease-IV, and Multi-Ethnic Study of Atherosclerosis studies DAMTCAII: Determination of Anthropometric Measures of Turkish Children and Adolescents, CASPIAN-IV: childhood and adolescence surveillance and prevention of adult non-communicable disease, MESA: Multi-Ethnic Study of Atherosclerosi

**Figure 3B:**
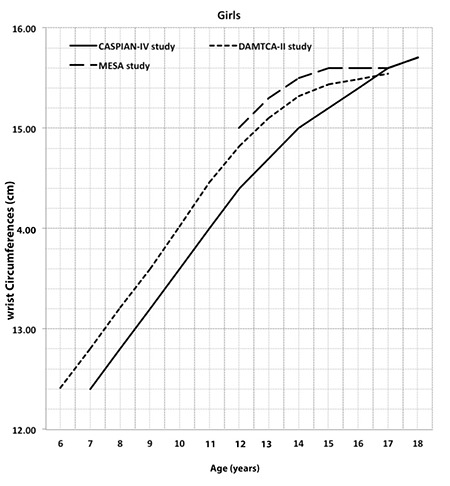
Comparison of 50^th^ percentiles of girls Wrist circumferences in Determination of Anthropometric Measures of Turkish Children and Adolescents-II, childhood and adolescence surveillance and prevention of adult non-communicable disease-IV, and Multi-Ethnic Study of Atherosclerosis studies DAMTCAII: Determination of Anthropometric Measures of Turkish Children and Adolescents, CASPIAN-IV: childhood and adolescence surveillance and prevention of adult non-communicable disease, MESA: Multi-Ethnic Study of Atherosclerosis
